# Motorcycles that See: Multifocal Stereo Vision Sensor for Advanced Safety Systems in Tilting Vehicles

**DOI:** 10.3390/s18010295

**Published:** 2018-01-19

**Authors:** Gustavo Gil, Giovanni Savino, Simone Piantini, Marco Pierini

**Affiliations:** 1 Dipartimento di Ingegneria Industriale, Università degli Studi di Firenze, Santa Marta 3, 50139 Firenze, Italy; giovanni.savino@unifi.it (G.S.); simone.piantini@unifi.it (S.P.); marco.pierini@unifi.it (M.P.); 2 Accident Research Centre, Monash University, Melbourne, 21 Alliance Lane, Clayton, VIC 3800, Australia

**Keywords:** stereo vision, stereo baseline, tilting vehicle, decalibration, motorcycle, ADAS, ARAS

## Abstract

Advanced driver assistance systems, ADAS, have shown the possibility to anticipate crash accidents and effectively assist road users in critical traffic situations. This is not the case for motorcyclists, in fact ADAS for motorcycles are still barely developed. Our aim was to study a camera-based sensor for the application of preventive safety in tilting vehicles. We identified two road conflict situations for which automotive remote sensors installed in a tilting vehicle are likely to fail in the identification of critical obstacles. Accordingly, we set two experiments conducted in real traffic conditions to test our stereo vision sensor. Our promising results support the application of this type of sensors for advanced motorcycle safety applications.

## 1. Introduction

Automotive sensors are key components of advanced active safety technologies for road vehicles. Research has shown that these technologies, named as Advanced Driver Assistance Systems (ADAS), are beneficial for the society as they contribute to mitigate, or even avoid, road crashes [1,2,3,4]. One of these technologies is Autonomous Emergency Braking (AEB), a system that detects imminent collisions and responds by automatically applying the brakes and slowing down the vehicle. AEB has been available on a range of passenger cars and heavy vehicles on the market for almost a decade and its effectiveness in the real world has been proven [1,3,4,5]. More recently, research has suggested that AEB could be effective also on other types of road vehicles, in particular motorcycles [6]. Such implementation of AEB on motorcycles would require that adequate sensing technologies are available also for tilting vehicles.

The quality of the remote sensing approaches allowing to artificially perceive the traffic scene for cars (e.g., Machine Vision, LIDARs, and RADARs) is the cornerstone of current ADAS. Regrettably, these sensors that are common in cars are not implemented in the market of tilting vehicles, not even in the high-end segment. And yet, the safety systems that such sensors enable could have an important impact, due to the high number of tilting vehicles in the fleets at global level [7,8,9]. Current mobility trends are in favor of the usage of tilting vehicles [10,11,12,13,14], partially thanks to their potential for: electrification, recyclability and air-quality improvement [15,16,17,18,19,20]. However, tilting vehicles are characterized by a high injury risk for their users, warranting the implementation of new and more effective safety technologies. Therefore the technological gap in the sensing technologies for tilting vehicles translates into a relevant safety gap [21,22,23].

The reason that causes the technological gap in tilting vehicles is that current automotive remote sensing sensors fail when they lean over [24]. This limitation owing to sensors’ design specifications explains the lack of equivalent ADAS for motorcycles. Previous studies of road safety reported range limitation in the use of these sensors on tilting vehicles [25,26]. The reason can be found in the roll angle fluctuations characterizing the dynamics of this type of vehicles, even when vehicles travelling straight.

To address these problems, we conceived a remote sensor designed for motorcycle safety system application. Our design is based on multifocal stereo vision to cover different regions of interest simultaneously, and to guarantee adequate depth accuracy to be part of future ADAS for motorcycles. The importance of this approach is to allow the use of artificial vision methods developed for ADAS in motorcycle safety. Consequently, we aim to provide the way to capitalize on relevant cutting-edge algorithms created for ADAS during the last 20 years and speed-up the development of needed motorcycle safety systems (or Advanced Riding Assistant Systems–ARAS) to make motorcycles and mopeds a safer means of transport. In particular, these stereo vision algorithms for vehicular application are successful in the following tasks: separate objects and surface structures by fast features extraction [27]; estimate the ground plane and perform a partial 3D reconstruction above it [28]; estimate the vertical road profile from the lateral projection of the 3D point cloud and model it as a clothoid [29]; classify surfaces on the road scene by the U-V Disparity concept [30]; perform obstacle detection in unstructured environment [31,32]; fuse stereo and optical flow to improve depth estimation accuracy, enabling fast detection of moving targets without classification processing [33]; model the scene by a polar occupancy grid [34]; generate a disparity map without perspective, simpler to analyze [35,36]; introduce a tracker filter to match the motion of a rigid point cloud with the kinematic model of a car, allowing to predict its immediate future location [37]; introduce a 3D perception primitive called “stixel” to capitalize on the depth information of almost all pixels of the image [38]; use dense disparity map to create digital elevation maps useful to detect important hazards for motorcyclist like traffic isles and small curbs [39]; trigger an autonomous emergency braking [40,41]; and employ different obstacle detection strategies in real-time [42].

The remainder of the paper is structured as follows: Section 2 explains the problems that imaging systems based on stereo vision technology need to overcome to be used as a part of a motorcycle safety system, and introduce several key concepts of stereo vision together whit the technical vocabulary used in the following sections. Section 3 presents the design of our custom build camera-sensors. We explain the operative considerations needed for the motorcycle application and how we accomplished them via stereo vision sensors. Section 4 explains the methodology to evaluate the sensor’s potential for motorcycle safety using two experiments. Section 5 presents the results obtained from the two experiments and their interpretation. In Section 6, we discuss the implication of this type of sensors to enhance safety in tilting vehicles, and we focus on technological sensor improvements from which the proposed remote sensor will benefit in the near future. Section 7 is a recapitulation of the main conclusions and lessons learned.

In addition, four appendixes to support and complement the core ideas of the publication. Appendix A presents the full parameters of our calibrated imaging sensors. Appendix B gives access to the online data corresponding to this research, such as the stereo image dataset and the 3D point clouds. Appendix C is an example that shows the importance of the online re-calibration for the motorcycle application. Appendix D briefly introduces the difficulties regarding the detection of narrow obstacles for other automotive remote sensors and repots some encouraging acquisitions realized for our system in dynamic scenarios.

## 2. Stereoscopic Vision Considerations for Motorcycle Safety Applications

A proper operation of stereo camera-based sensors requires the use of two synchronized cameras as a whole, which is achieved through the jointly characterization of them (the stereo camera calibration). An invariant calibration of the 3D sensor would assume constant parameters of the imagining system, such as relative distance and orientation between the two cameras, which are physically determined by the location of the cameras in the stereo rig.

Regrettably, the assumption is not valid for the motorcycle application due to the deformation (micro-bending) of the stereo rig. A motorcycle, as a lightweight vehicle, has less potential than a car to damp the vibrations generated from road irregularities. In fact, in normal riding conditions the vehicle frame is subjected to intense shocks, which passes to the stereo camera rig, producing dynamical changes in the instantaneous distance and orientation between the cameras. As a consequence, an invariant stereo calibration is not suitable for a moving motorcycle setup. In addition, the optical zoom of the long range cameras makes common mechanical anti-vibration solutions ineffective. However, one possible solution is online stereo re-calibration.

### 2.1. Stereo Vision Fundamentals

Concepts of stereo vision considered relevant for the design and implementation of our multifocal stereo camera sensor are recapitulated hereafter. More details on stereo vision and 3D geometrical modeling principles can be found in [43,44].

Estimating depth from stereo imaging is a triangulation task. In the human vision, to solve this task visual information derived from two eyes is used to estimate depth from the so called binocular disparities [45]. The disparity is the parallax observed between corresponding world-points (in 3D space), and it is inversely proportional to the distance from the sensor viewpoint (*Z*, see Equation (1)).

If we consider the stereo triangulation in epipolar geometry, the correspondence of points between two images is obtained by means of imaginary scan lines. The distances in pixels along scan lines are the disparities between couples of correspondent points. Epipolar geometry defines epipoles and epipolar lines. For each camera and each point in space, the epipole is defined as the intersection of the camera imager and the line passing from that given point and the focal point of the camera. The line in the space connecting two corresponding epipoles of the two cameras is the epipolar line. Finally, epipoles and epipolar lines have a representation in a rectified space computed using the fundamental matrix [46,47], thus generating rectified images (after lens distortion correction).

Epipolar lines in rectified images are horizontally aligned. This characteristic simplifies the search for matching correspondent features to a simple search within image rows between the pair of rectified images [48]. For real time computation, a suitable stereo correspondence method is the Semi-Global Matching algorithm [49].

In our setup, both cameras are assumed to have the same focal length *f* expressed pixels unit. The distance between the cameras is their baseline *b* in distance unit. The difference of the relative projection of a world-point is the disparity *d*, generally expressed in terms of pixel units. Resulting depth can be computed using Equation (1), which shows that the disparity is inversely proportional to the distance *Z* (expressed in distance units) of the object.

(1) $$Z=\frac{f\cdot b}{d}$$

### 2.2. Field of View and Depth of Field

The Field of View (*FoV*) of a single camera is a solid angle through which the imaging sensor is sensitive to the light. Therefore, the *FoV* define the periphery of the 3D volume of the inspection captured on the camera imager sensor. The *FoV* depends on a combination between the size of the imager and the camera lens. Therefore, the focal length defines the *FoV* (Equation (2)), which is related to the focal length *f* (in distance units) and the horizontal size of the imager *h* (in distance units).

(2) $$FoV\left[{}^{\circ}\right]=2\cdot \tan^{-1}\left(\frac{h}{2\cdot f}\right)$$

Lenses with a fixed focal length are designed to be focused for different distances but at expenses of less quantity of light from the scene imaged (brightness). Therefore, in the case of a multifocal strategy it is recommended to use fixed lenses that select a limited Depth of Field (selective focus along the depth axis) in the desired depth range of measurement, in order to maximize the light sensed from this part of the scene.

### 2.3. Caracteristics of a Stereo Camera Rig: Common FoV, Range Field and Horpter d = 10

**Figure 1:**
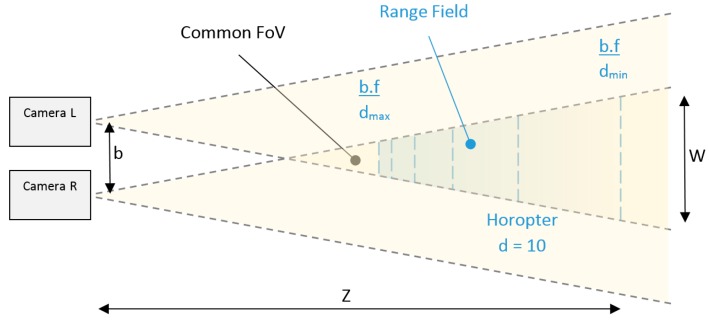
Top view of a conventional stereo vision system. Detail of the Range Field including the boundary of depth triangulation range adopted at the Horopter of 10 disparities.

Rectangular imager sensors modifies the concept of circular *FoV*, as a consequence, it is specified as a Diagonal *FoV*, Vertical *FoV* and Horizontal *FoV*. Additionally, in this paper common *FoV* is defined as the overlap between the *FoV*s of the pair of cameras on a stereo rig. The common *FoV* between the left and right cameras defines the lateral boundaries (vertical and horizontal) of the Range Field (Figure 1). Employing fixed lenses (*b.f* = constant) the possible depth range to perform triangulation is defined for the range of disparities (*d*_min_ and *d*_max_), which determines the rear and frontal boundaries of the Range Field.

The previous top view representation of the Range Field (Figure 1) is a simplification of a 3D volume termed frustum, which defines the Range Field. Thus, it is possible to determine the depth information for all objects inside this frustum. The Range Field was shortened by the transversal surface defined as Horopter of 10 disparities, in order to neglect the bias error of depth and satisfy the real-time constraints of our application.

### 2.4. Depth Triangulation Error in Stereo Camera Sensors: Case of Long-Range Applications While Moving

The triangulation error (Δ*Z*) in a stereo system is defined according to Equation (3), for which *Z* is the depth in world coordinate frame and Δ*Z* is the depth error in distance units. The value Δ*d* (disparity step) is directly related to the depth error and this component of the error depends on the capabilities of the stereo matching algorithm for achieve sub-pixel refinement.

(3) $$\Delta Z=\frac{Z^{2}}{f\cdot b}\cdot \Delta d$$

In stationary stereo measurements, the triangulation error follows a normal distribution [50,51,52,53]. However range bias error is induced for the camera position [54,55,56] and depending the application researchers did or did not neglect it. Thus, the range bias error in moving systems need to be considered different from the Gaussian distribution. For this reason, in Simultaneous Localization and Mapping (SLAM) applications in order to ensure robustness, the maximum depth triangulation is defined a priori until a maximum distance 40 times the baseline [57]. This limit adopted for SLAM (mapping needs) is very conservative for long-range stereo, for example our long-range baseline is duplicating this relationship (depth range 80 times the baseline) while keeping the maximum depth error below 3% (more details in Section 3.3).

Research of long-range stereo applications have quantified the nature of depth stereo error. In their research, an experimental setup was conceived for tracking distant features with sub-pixel accuracy. They showed that the probability density distribution of the depth measurement error is non-Gaussian [58]. In fact, the distribution is skewed and presents a long tail [58]. This produces an effect of over estimation of the triangulated position that increases with the distance. Other research pointed out the possibility of correcting the bias error of depth in the lower values of disparity, for integer disparities calculations [59].

### 2.5. Sub-Pixel Accuracy and Relationship with Depth Accuracy: Case of Car Detection

Digital images are limited to pixel resolution because the objects in an image are spatially quantized at the resolution of the imager. However, the edges of the real object cannot necessarily be sensed for the entire pixel of the imager. In this cases, a more accurate object location must be defined in fractions of pixel. This situation is referred as sub-pixel resolution, which is common to encounter for far objects imaged [60].

From Equation (3), it is clear that a disparity value (*Δd*) inferior to one will decrease the triangulation error, or for the same error the triangulation Range Field can be extended. On the other hand, a fractional value of disparity can be seen as virtual expansion of the baseline (*b*). In this second regard of fractional *Δd*, for example, a portable stereo sensor designed to assist the visually impaired has achieved 1/8 sub-pixel resolution in an embedded system, compensating for the short baseline allowed for the wearable application [61].

In the automotive field, the first application of this concept to car detection reported an empirical limit in 1/4 of sub-pixel accuracy by employing a quadratic interpolation [62], which is a simple constant-time operation suitable for real-time implementations. Beyond this empirical limit of sub-pixel accuracy, the car depth triangulation is not robust due to the pixel-locking effect [63,64]. Subsequent experiments endorsed the 1/3 or 1/4 as a robust sub-accuracy measurement on road traffic scenarios [65,66], but they extended the measurement case to texture-less regions [66], which is a big challenge for stereo matching algorithms.

A recent publication [67] reports the achievement of 1/5 of sub-pixel accuracy highlighting the importance of the census transform [68,69,70,71] to provide a robust stereo matching. The robust matching function “census” was also pointed out in a previous analysis of sub-pixel decalibration error [72]. Nowadays, the census transform is recognized as a noise-robust stereo matching. It is used to provide proper disparity maps during the training of machine learning algorithms in applications of 3D understanding [73].

For the remote sensor, we considered HD imagers (resolution 1280 × 720) to set an equivalent value of 1/2 of sub-pixel accuracy with respect to the imagers in the aforementioned literature. This conservative decision limits the full range of the measurement at expenses of a gain in robustness against sub-pixel camera decalibration (calibration loss).

### 2.6. Camera Decalibration (calibration loss)

Depth triangulation in stereo camera systems depends critically on accurate calibration of each camera pair and in a vibration-free set up. The calibration is constituted by intrinsic and extrinsic parameters, for which the latest refers to the relative camera pose (3D orientation) between the two cameras. The extrinsic calibration depends on the physical fixation of the pair of cameras during time. In this regard, a motorcycle is a harsh environment where vibrations coming from the engine (which is rigidly fixed to the motorcycle frame), the vehicle-road interaction, and aerodynamic drag forces can slightly modify the instantaneous pose between the camera pair along time (that cannot be seen with the naked eye).

Depending on the application, a variety of techniques are used to solve this issue. Examples: (a) In visual odometry it is usual to perform continuous stereo extrinsic re-calibration (5 Degrees of Freedom, DoF) operating on sparse stereo correspondences on stereo frame basis [74]; (b) In mapping applications the re-calibration is 6 DoF between the cameras in the way of visual odometry but with the addition of GPS information [75]; (c) In low altitude aerial imagery (< 30 m), the modal deflection of the drone wingspan is monitored which accelerometers in the tip of the wings were the cameras are located, using this information to compensate the relative angle of the stereo pair [76]; (d) In satellite imagery the undamped micro-vibrations on the satellite are software-compensated by the measures realized over known flat points in the earth [77]; (e) In areal imagery, a tailored bundle adjustment technique is used to refine camera parameters achieved altitude operations up to 120 m employing a wide baseline [78]; (f) Automated driving have also bundle adjustment implementations in which they estimate online both extrinsic and intrinsic camera parameters with a pre-definition of the scale [79]; (g) A recent approach for robotic applications computes 5 DoF of extrinsic by a marker-less nonlinear optimization method [80]; and (h) In “motion stereo” or Structure-from-Motion (SfM) applications a relaxation of the epipolar constraint is performed. In these cases, the stereo frame is generated for a monocular moving camera, which moves over a rigid scene. The main assumption of this technique is a small vertical displacement, and consequently the matching strategy is relaxed by exploring a corridor around the epipolar line [81].

### 2.7. Stereo Confidence Clues

The results corresponding to the Disparity Map (DM) calculation may have associate a level of confidence meaning that, for each pixel of the DM a probability that express how real is the triangulation can be associated. Non-real triangulations due to lighting reflections or circumstances of bad visibility, like rainy weather, can lead to wrong detections. Several metrics were developed as a way of quantifying the stereo confidence; a first framework for stereo confidence clue evaluation defined a taxonomy [82] that was adopted for the research community to this end.

The stereo confidences is a valid measure that can be used in absence of ground truth data [83]. This is important because rendering artificial scenarios that contain realistic outdoor adverse situations, and realistic erroneous sensor data, is a huge challenge [82,84]. Therefore, even the advantage of the ground truth of synthetic imagery is not a practical approach to use for this case. The practical approach consists in using real imagery acquired in adverse situations and human annotations or labeling of the DM based on the visual information [85]. The tedious approach of binary labeling the confidence zones, allows to implement a Bayesian inference that is better to assess confidence metrics because it does not use just the annotations in the images.

The variety of stereo confidence metrics perform differently in varying outdoor conditions, thus the wise fusion of them implies more robustness against stereo matching errors. Machine-learning approaches allow to use a set of metrics to improve the accuracy of the stereo confidences [86,87,88,89]. Recently, machine-learning approaches to big stereo data collected in adverse weather allowed for a self-supervised strategy that automatically labels confidence zones effectively [90].

## 3. Materials

We provide a detailed explanation of the design considerations of our camera-based remote sensors and consequently, how the system was evaluated for the application in motorcycle safety.

### 3.1. Sensor Architecture (Multifocal Stereo Rig and Processing)

**Figure 2:**
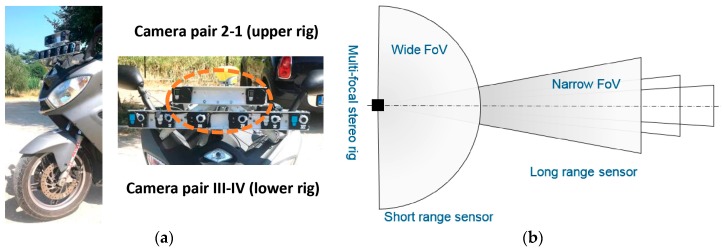
Overview of the imaging system. (**a**) Multi-focal stereo rigs installed in the frontal part of the vehicle and fixed to the scooter frame; (**b**) Top view of the 3D space to measure in front of the scooter (the outer stereo cameras are used for development purposes and future extension of the long range of measurement).

The multi-focal stereo rig is shown in Figure 2a. It is composed by 8 low-cost cameras with fixed focal lenses conforming 4 stereo camera pairs. All cameras have a rolling shutter imager sensor with HD resolution (1280 × 720).

**Figure 3:**
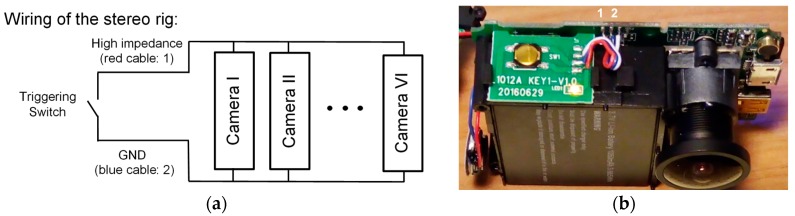
Wiring detail for the synchronization of the six cameras. (**a**) Circuital scheme; (**b**) A disassembled camera showing the location of the electrical connections labeled “1” and “2”.

The synchronization between the six cameras (Camkong) of the lower rig was performed by hardware (Figure 3), while the two cameras (GoPro Hero Black) of the upper rig used wireless parring. In this publication are used only the central cameras corresponding to the camera pair III-IV (short-range sensor < 8 m) and the camera pair 2-1 (long-range sensor < 22 m) as indicated in Figure 2a. The other cameras are installed for development process.

The design of the remote sensor initially requires to define the spatial zones at the front of the scooter which are necessary to scan, as it is shown in Figure 2b. The depth range of these zones need to be defined according to the highest traveling speed allowed for the scooter and the possible colliding car. The application focusing on the urban scenario were top speed is restricted to 50 km/h.

Applications in advanced safety systems like the conceptual Motorcycle Autonomous Emergency Braking (M-AEB), requires a precise triggering to avoid false positives. In particular, for M-AEB safety system, the depth resolution required for the proper identification of the inevitable collision state was defined in a spatial grid of 20 cm [91]. Thus, our remote sensor target this specification.

The short-range stereo pair have fisheye lenses to scan a wide 3D space ahead of the vehicle. The light arrives to the imager from multiple directions, these motivated to define a short Range Field for the stereo triangulation. The case of the long-range cameras differ, because the narrow Field of View (*FoV*) of the lenses focalize the scanning volume in a narrow frustum.

Consequently, we selected lenses through their *FoV*s. Next, the baselines for the two pair of cameras that allow having a common Depth of Field (DoF) enclosing the desired frustum, as shown in Figure 2b. Additionally, to ensure sharp images to be captured in the range of the sensor (e.g., short- or long-range) with the aim of performing the stereo triangulation, a trade-off between the *FoV* and the focal length was chosen (Table 1).

After that, the 3D space measured is confined in a frustum, which is defined by the common *FoV* and the disparity range determining the Range Field of the stereo sensor (Figure 1). Regarding the Range Field of our sensors, they have to be included on the Depth of Field of each camera. In our application we decided to shorten Range Field as a manner to warranty repetitively at the full range of the measured space in a robust manner.

For the initial definition of the Range Field, the adoption of a Horopter of 10 disparities for the farthest measure was motivated to neglect the bias error of depth. At disparities on the order of 10 pixels or less, the effect of the non-Gaussian error in depth cannot be negligible for the triangulation calculation [58]. The Table 2 shown the values calculated using the Equation (1) for our specific application.

The depth discretization is not linearly distributed inside the frustum, as illustrated in Figure 1 by the parallel lines (top view of parallel planes). Thus, the largest and the shortest depth discretization are defined by the last and first two Horopters (depth planes) of the Range Field.

Considering the requirements for motorcycle safety systems, a depth grid of 20 cm is required by the conceptual M-AEB. The Table 2 shows the potential of the designed sensor. For example, when the long-range sensor (Camera pair 2-1) is measuring 18.93 m ahead the sensor, the depth discretization using ¼ of sub-pixel accuracy is 19 cm. When the obstacle is approaching, the depth discretization become even smaller offering more depth accuracy.

Remark: in some Disparity Maps showed in this paper, the reader may find different range of disparities of that required for M-AEB (Table 2), this is only for better visualization in the paper.

### 3.2. Calibration of the Multi-Focal Stereo Camera Sensors

The procedure allowed the calibration of all the cameras of the stereo rigs, it means obtaining all the intrinsic and extrinsic parameters that are used to model the camera and its pose in the space. In [92] is presented and explained the first method that allowed to calibrate a 3D imaging sensor with an inexpensive planar calibration pattern. The intrinsic and extrinsic parameters of the camera mathematical model are also descripted in this important paper.

Essentially, we moved the checkerboard specified in [93] throughout the common Field of View (*FoV*) of all stereo pairs that were recording video concurrently. Consequently, the analysis of the footages of each camera allowed to picking up the suitable stereo frames (these that contain a complete view of the checkerboard in the couple of images that conform the stereo frame) to perform the stereo calibration for each pair of cameras.

Next, we started the corner detection process, searching for symmetrical corner features in the images in order to find the checkerboard. State-of-the-art subpixel accuracy algorithms [94] contributed to obtain a proper calibration of our remote sensor. During the calibration process, we selected the frames for which the reprojection error was below to a low threshold empirically selected. To conclude, we calculate the two-step nonlinear optimization needed [95] to get the camera calibration parameters.

**Figure 4:**
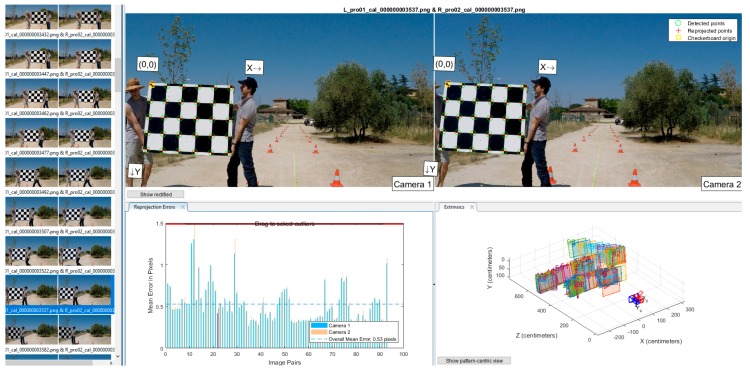
Overview of the graphical user interface of the Matlab Application for the stereo camera calibration.

In Figure 4, the calibration procedure illustrated by an example that corresponds to the long-range stereo camera sensor. The procedure is the same for each stereo camera pair. The thumbnails on the left shows a set of stereo frames employed for the calibration. Each thumbnail is labeled with the file name and extension for reference of the files used.

On the top of Figure 4, the pictures labeled “Camera 1” and “Camera 2” have overlaid the corners detected on the checkerboard in the stereo frame. On the bottom part, the bar plot depicts the calibration accuracy for each stereo frame in pixel units (reprojection flap). The reprojection error is the distance in pixels of the location of corner features detected in each original picture used during the calibration with respect to the location of the same corner features in the rectified image. The rectified image is calculated based on the set of camera parameters obtained in the calibration. Thus, differences will exist (the reprojection error in pixels) because the calibration parameters tends to satisfice the calibration of all the set of images simultaneously.

Finally, on the bottom right of Figure 4 is shown a 3D diagram that represents a three-dimensional volume with units in centimeters (extrinsics flap). This volume contain several planar rectangles labeled with numbers. Each of the rectangles are placed in different locations and with different pose (3D orientation in the space). Notably, each rectangle represents the location and pose in the space of the marker (checkerboard) during the calibration process.

The main camera parameters obtained as a result of the static camera calibration performed for both stereo sensors are shown in Table 3, while more detailed information in the Appendix A.

### 3.3. Determination of the Range Field (Verification of Desired Depth Accuracy)

The design considerations of the imagining system developed, mainly in terms of focal lenses, common *FoV*s, baselines, resolution of the imagers, proportioned a baseline with the ideal measurement capabilities of the stereo vision sensors. However, several practical factors can affect the measurement range of a stereo camera system and this experiment was designed to quantify the Range Field of all the stereo camera rigs.

**Figure 5:**
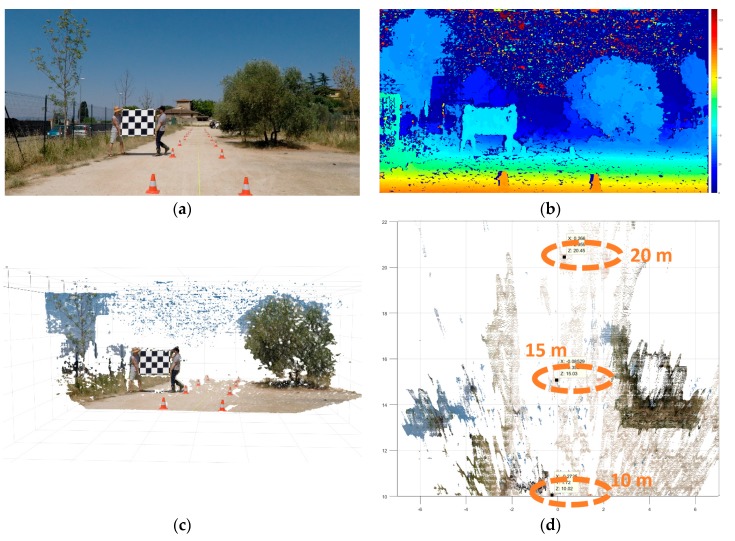
Depth accuracy quantification of the calibrated stereo camera (long-range camera rig). (**a**) Rectified left picture acquired by the long range stereo camera sensor; (**b**) Disparity Map of the scene (range from 0 to 128d); (**c**) Three-dimensional reconstruction or 3D point cloud of the scene imaged; (**d**) Top view of the 3D point cloud highlighting the location of the traffic cones originally placed at 10 m, 15 m, and 20 m. This 3D point cloud can be download according to Appendix B for a better assessment of the reader.

The test was conducted in an open and flat surface after having delimited a rectilinear corridor by traffic cones (Figure 5a). The corridor had 2 m of wide and 45 m of length, and it was defined by cones of 30 cm height placed in couples spaced 5 m. The nearest couple of cones are located a 5 m of our sensor. We re-use the same stereo videos recorded during the camera calibration process, for this reason two people appear holding a checkerboard. In the Disparity Map (DM) of Figure 5b, we employ the planar surface of the checkerboard to assess the homogeneity of the disparities calculated in function of the depth.

The 3D reconstruction of the scene imaged (Figure 5c) shows the capability of the remote sensing approach to measure the 3D space. The 3D point cloud was calculated for a three-dimensional space starting from 5 m to 30 m of depth for development purposes and the definition of the Range Field.

The point cloud calculations are not supposed for a real time application, it is only used for helping to assess the quality of the 3D information measured. For the top view of the scene (Figure 5d) the point cloud was calculated from 10 m to 22 m which corresponds with the Range Field of the long-range sensor. We highlighted the location of the 2nd, 3th and 4th couple of cones corresponding to the depth distance of 10 m, 15 m, and 20 m.

**Figure 6:**
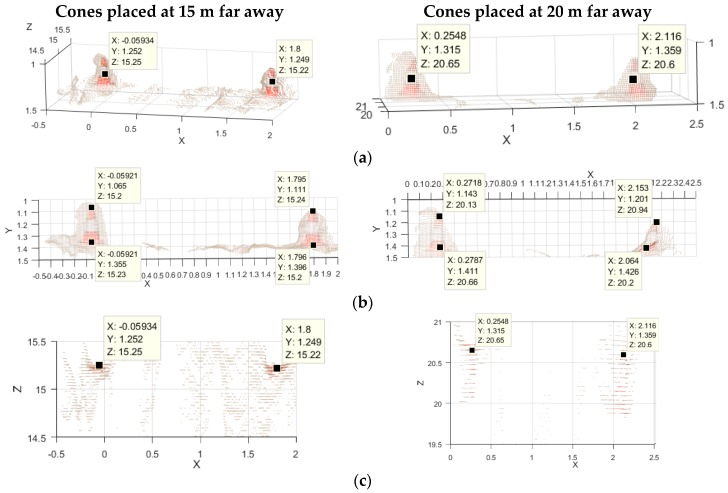
Analysis of small narrow objects in the farthest half of the Range Field (long-range camera sensor). 3D control points were located at similar places for each couple of cones for the analysis. (**a**) Detail of the 3D representation of the targets; (**b**) Frontal view of the targets (grid sized 10 cm); (**c**) Top view of the 3D point clouds (grid sized 50 cm). For the cones at 20 m the fattening effect becomes evident (depth artifact).

The bias error of depth grows for longer distances as is expected for Equation (2), this is being negligible for the first two pairs of cones and a tolerable 1.68% and 2.25% systematic errors (more details in Figure 6). We define our systematic depth errors as a tolerable because even without the proper error cancelation they are inferior to 3%, this value that was found as a requirement for reliable driving assistance functions in cars [96].

### 3.4. Determination of the Horizontal Resolution of the Stereo Vision Sensor

In order to quantify the horizontal resolution of the stereo vision system we carefully located objects of known dimensions, like traffic cones of 30 cm of height separated 2 m, inside the Range Field of the stereo camera sensor. In Figure 6 are depicted three different views of the measurement corresponding to the couple of cones located a 15 m and 20 m. From the measured values it can be seen that the horizontal measurement of the targets present a systematic error about 7% to 8% from the two measures.

Therefore, the 20 cm of horizontal resolution required for the Motorcycle Autonomous Emergency Braking (M-AEB) application [91] can be achieved for our stereo camera sensor. Additionally, this the horizontal resolution have the potential to be used in for the detection of small road hazards strategies [97].

### 3.5. Camera Online Re-Calibration

The vibrations in the scooter temporally misalign the cameras of the calibrated stereo camera sensor (decalibration). Therefore, an online re-calibration is needed to perform triangulation. The re-calibration implemented is based on a two-step rectification under two assumptions:

- Invariance of intrinsic parameters of each camera.
- The extrinsic parameters of each camera pair varies within a small range.

In this way, the images are rectified two times to avoid problems of scaling. The first time uses the camera parameters obtained in the static calibration (Appendix A), and a second time uses the sparse pixel image correspondences (rectification tuning).

Nevertheless, as multiple correspondence measures are available and matching methods can lead to significant differences in matching results, there is a trade-off between execution time and descriptor quality to be evaluated empirically [98,99,100,101,102,103,104] for each application case.

As starting step, we decided to perform a qualitative comparison by employing two different kind of keypoint descriptors, a histogram-based descriptor and a binary descriptor. It is worth saying that the two feature descriptors chosen have shown good performance in real-time implementations (comparison in Appendix C). We excluded correlation methods of our comparison due to its high computational complexity [105].

The election of SURF (Speeded-Up Robust Features) method [106,107] as image feature descriptor and extractor was because it is a robust [102] and quick [98] keypoint descriptor [104]. These characteristics make it suitable for the self-calibration of wide baseline stereo camera systems.

**Figure 7:**
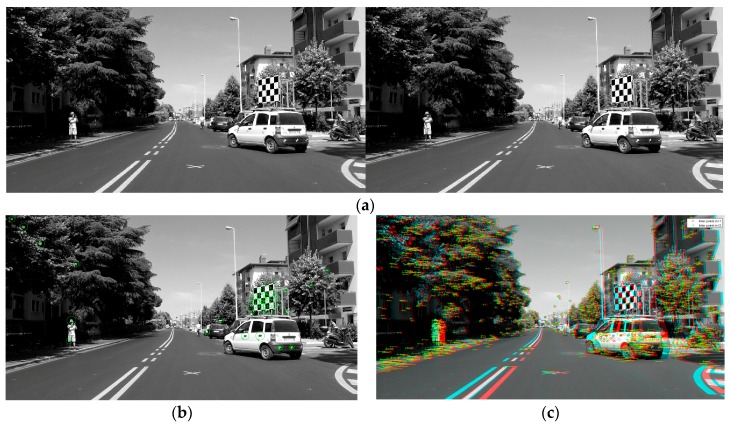
Illustration of the automatic camera extrinsic parameters re-calibration. (**a**) An initial rectification of the stereo frame according to the static calibration values; (**b**) SURF feature extraction in both images of the stereo pair (circle’s diameter represents the scale of the feature); (**c**) The salient features matched (correct pixel assignments indicated by yellow connections) are overlaid on a 3D anaglyph.

In Figure 7 is illustrated the procedure for the second step of the re-calibration implemented. First, calculate sparse point correspondences between the images rectified to identify 3D salient points of the scene (Figure 7b). Compute salient points per image with the point descriptor SURF. Next, putatively match the correspondent features between the images (Figure 7c) in order to estimate the “fundamental matrix” [43,44] thanks to the RANSAC (RANdom SAmple Consensus) method. Thus rectifying the images, it means, aligning the images such that corresponding points will appear on the same rows in both new images to perform triangulation.

Up to now, we implement the online camera re-calibration employing SURF features and SURF feature descriptors. However, binary descriptors are more suitable for real-time applications because they requires significantly less memory than histogram-based descriptors like SURF. This motivated our second re-calibration test employing SURF features and FREAK (Fast REtinA Keypoint) descriptors. FREAK is based in human retina behavior [101].

A real example of dynamic decalibration acquired from our stereo camera rig is presented in Appendix C next to the results of the two re-calibrations implemented. In addition, we provide an image dataset with ground truth corresponding to a trial of the pre-crash experiment. The dataset is provided through an online repository and the ground truth can be obtained from the satellite marker on the colliding car [93].

## 4. Test Protocols

We designed two experiments to assess the capability of our custom built remote sensors to perform depth and orientation measurements of target cars in a real traffic scene. All test were conducted in outdoor and clear visibility conditions, being the most common circumstance of motorcycle crashes in Italy (according to national statistics for the period 2000–2012 [9,24]). The experiments were conducted under sunlight to face the challenge of dealing with reflective and semi-transparent surfaces that can affect the capabilities of optical measurement systems. In addition, we performed our experiments on public roads and with normal traffic, to observe realistic traffic situations and assess the behavior in such setting (approved by the ethical committee of the University of Florence).

The first experiment consisted in a turning maneuver. It is known that automotive remote sensors are not designed to operate when the instrumented vehicle is tilted and in such conditions they would fail [24]. This experiment aimed at measuring stationary target cars while the instrumented scooter was negotiating a curve in an intersection, while tilting with roll angle greater than 8 degrees.

The second experiment analyzed a pre-crash scenario (pre-crash test) and consisted in emulating the trajectories of a scooter and a car that led to a documented crash [108,109]. The aim of this experiment was to assess whether the sensors under test can sense the changing traffic scene at least 1 s before the collision becomes inevitable. The experiment took place in the physical location where the accident had happened and emulated the vehicle trajectories before the actual collision event employing instrumented vehicles.

The details of the test protocol and the instrumentation of the vehicles were described in a previous paper [110]. In addition to the sensors equipping the vehicles, we used a checkerboard mounted on the top of the target car to retrieve ground truth of the car pose (3D orientation in the space) from the imaging system at frame level, thanks to the satellite marker method [93].

## 5. Results

The following results for each of the two experiments are presented in the form of Disparity Map (DM) and 3D point cloud (3D reconstruction).

### 5.1. First Experiment Involving Tilting Dynamics: The Turning Maneuver

**Figure 8:**
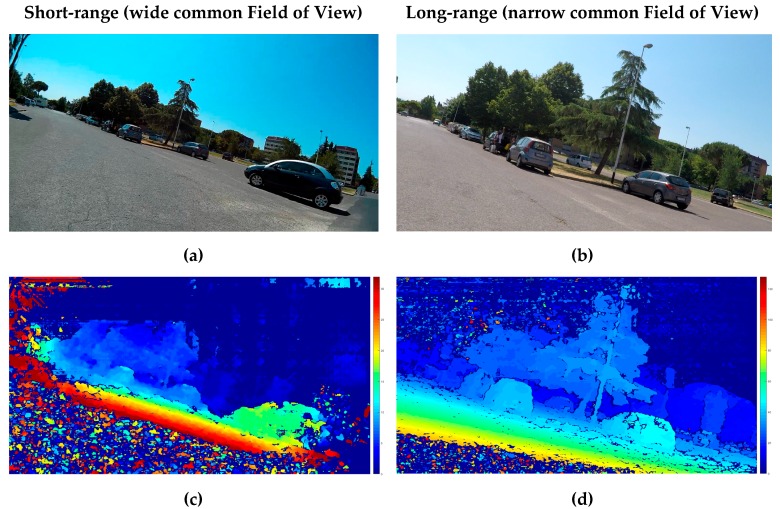
Analysis of a turning maneuver: measurement of the space in front of the scooter. Short-range and long-range measurements are depicted simultaneously for comparison. (**a**) Rectified left picture of the short range stereo camera sensor; (**b**) Rectified left picture of the long range stereo camera sensor; (**c**) Short-range Disparity Map (0 to 32d); (**d**) Long-range Disparity Map (0 to 128d). The 3D point cloud is available for download (Appendix B).

**Figure 9:**
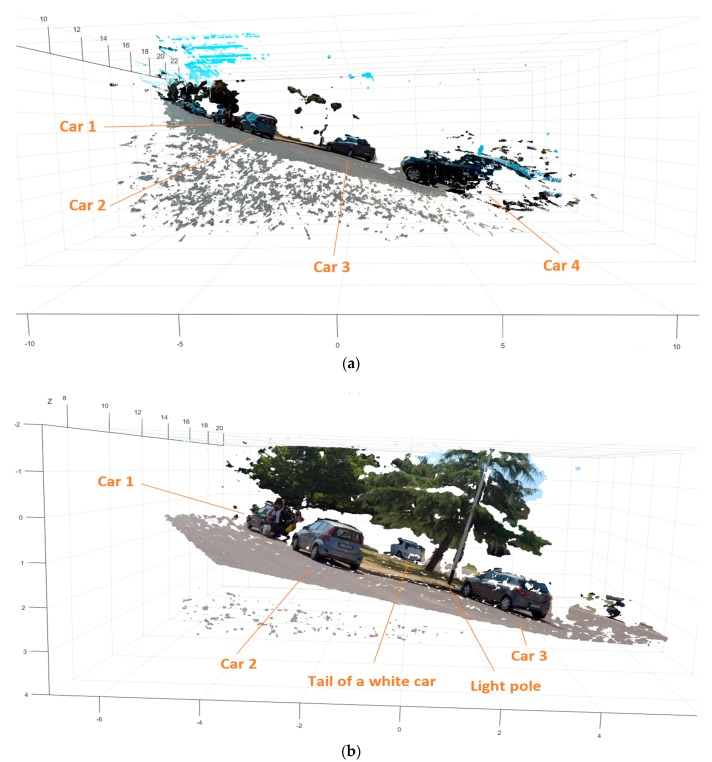
3D point clouds corresponding to the turning maneuver scene calculated from the information provided by the two stereo camera sensors. (**a**) Reconstruction for the short-range stereo camera (wide common Field of View); (**b**) Reconstruction for the long-range stereo camera (narrow common Field of View).

**Figure 10:**
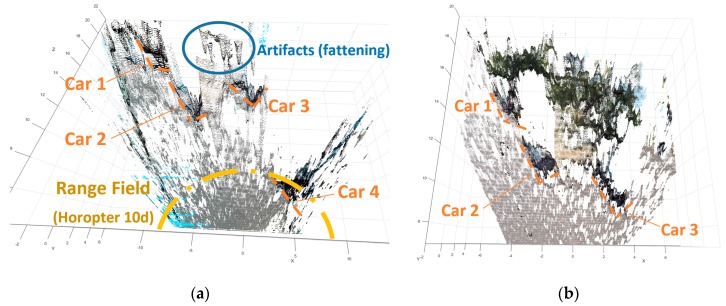
Top view of the 3D point clouds corresponding to the turning maneuver. (**a**) Depth measurement delivered by the short range sensor (accurate depth measures are inside the Range Field); (**b**) Depth measures delivered by the long range sensor (Car 4 is not in the common Field of View of this stereo camera sensor).

The following results (Figure 8, Figure 9 and Figure 10) correspond to the case in which the scooter is executing a turning maneuver at an intersection. In this situation the scooter is tilted more than 13 degrees, excluding the successful use of traditional automotive remote sensing sensors [24].

Figure 8 presents the traffic scene sensed by the short and long range sensor and the associated DMs. With regards to the parked cars visible in the scene, which did not move throughout the experiment, the measurements in repeated trials presented similar information. This condition allowed us to compute similar depth measurements of the static scene.

Both DMs depicted in the figure show the accurate detection of the ground, which is used to define a ground plane and from that, the tilted Region of Interest (RoI) for the subsequent analysis. In addition, the different range values contained in the DMs are consistent with the type of lenses and baselines employed for the short- and long-range sensing, respectively.

In the short-range sensor (the shorter baseline of the stereo camera rig), the fisheye lenses used embraced the light coming from a wide volume of the 3D space into the imaging sensors. Consequently, the maximum value of the disparity becomes big (see Table 2). In Figure 8c DMs were represented using a color scale of 32d instead of 128d (see Table 2) to highlight the presence of a car (car 4) that was not visible in the long range view.

In the long-range sensor, the more directional type of lenses (narrow *FoV*) and longer baseline of the stereo camera rig produced a smaller range of values of the disparities (see Table 2). This is a consequence of measuring a smaller part of the 3D space with an imager of the same size, thus providing more depth discrimination of the 3D space.

**Figure 11:**
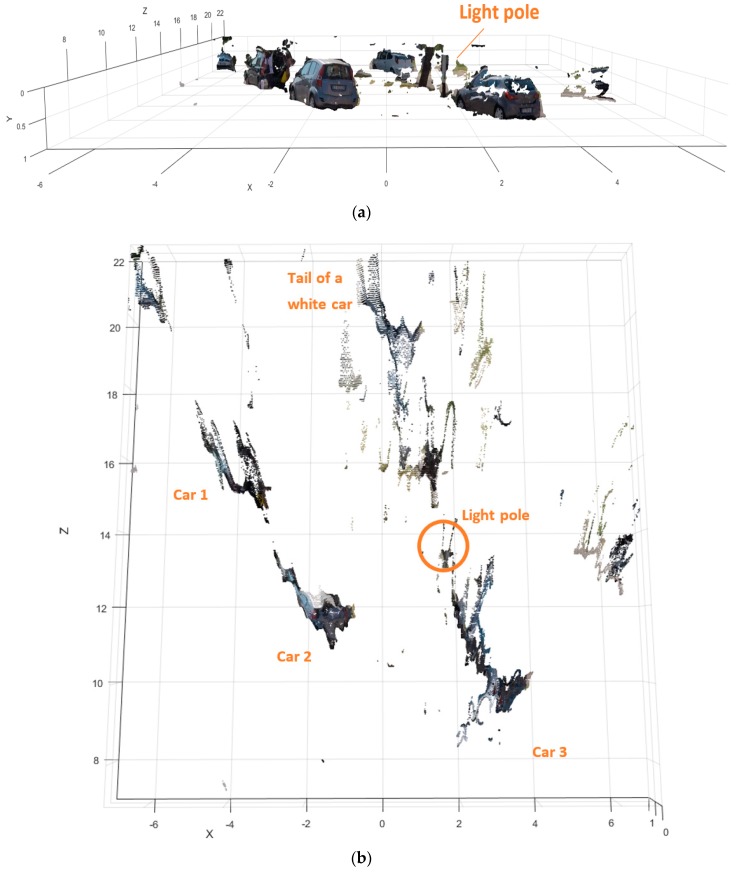
Cleaned measurements of the corresponding to the turning maneuver. (**a**) The 3D point cloud inclined 13° to compensate the leaning of the scooter; (**b**) Top view of the measures.

Figure 9a shows the 3D point cloud of the 4 target cars measured by the short-range sensor (manually labelled). In Figure 9b shows the long-range sensor measures of the same scene. Only 3 of the previous target cars are visible, because of the narrower *FoV* of this sensor. In addition to this, it is worth noticing the tail of an additional white car visible behind the trees at the boundary of the Range Field (Figure 8b,d, Figure 9b and Figure 11b).

In Figure 10, the depth measurement performed for the imagining system (short- and long-range) are presented in more detail. In both measurements cars 1, 2, and 3 can be identified. However, only the depth measure delivered by the long-range sensor (Figure 10b) is reliable. In fact, these cars were visible in both sensors, but they were located inside the Range Field of the long-range sensor only. As mentioned before, the condition that the targets are inside the boundaries for the triangulation (horopter of 10 disparities) ensures the possibility to neglect the influence of the non-Gaussian error in the DM. Otherwise, such error should be taken into account for proper depth triangulation [58].

Considering Figure 10a, car 4 is properly sensed due to the wide common FoV of the short-range sensor. Additionally, in the same figure artifacts are present outside the Field Range, in the low values of disparities. At very low disparity values, the erroneous matchings of the SGM (Semi-Global Matching algorithm) [49] generates fattening, which is a matching error amplified by the depth discretization for the pixel accuracy resolution (including subpixel accuracy). Fattening are common artifacts in outdoor stereo vision, and post-processing approaches that remove them in 3D mapping applications are not a real-time task.

Note: the 3D point clouds presented until now contain all the raw data measurements retrieved from the stereo cameras. However, certain details are difficult to assess the 3D measurements. Therefore, in order to simplify the assessment of the quality of the measures corresponding to the long-range sensor, we will “clean up” the 3D point cloud. The cleaning process consisted in two steps:

- To post process the three-dimensional representation by removing the 13 degrees of inclination of the scene without altering the quality of the 3D reconstruction.
- To extract the points that lies outside of a Region of Interest (RoI).

In this manner, we extracted and inspected a RoI above the ground plane as it can be seen in Figure 11a, where the detection of a narrow object (light pole) is highlighted.

In Figure 11b the top view of the clean imaged scene is presented, with the reference to the vehicles and the light pole location.

The results presented in Figure 11 illustrate the measurement capability of the sensor to measure the sides of the parked vehicles, even under large inclination of the sensor (13 degrees), where other automotive remote sensor technologies cannot deal with [24]. This capability of the proposed remote sensing approach enables the utilization of the obstacle detection, tracking, and depth perception during the normal operation of a tilting vehicle.

The proposed remote sensing strategy was designed to handle changes of orientation (tilting) of the sensor by exploiting the wide diagonal *FoV* and the generous horizontal and vertical spatial discrimination offered by camera-based sensors. These two key design specifications allowed us to measure the relevant depth information of the traffic scene while the remote sensor was subjected to the dynamics of a tilting vehicle (tilted 13 degrees), as was never reported before.

### 5.2. Pre-Crash Test (Based in a Real Motorcycle Crash)

In a previous publication [110] was defined a methodology to assess the possibility of computing accurate triggering for a conceptual Advanced Rider Assistance System (ARAS) in realistic pre-crash conditions. The ARAS was the Motorcycle Autonomous Emergency Braking (M-AEB).

**Figure 12:**
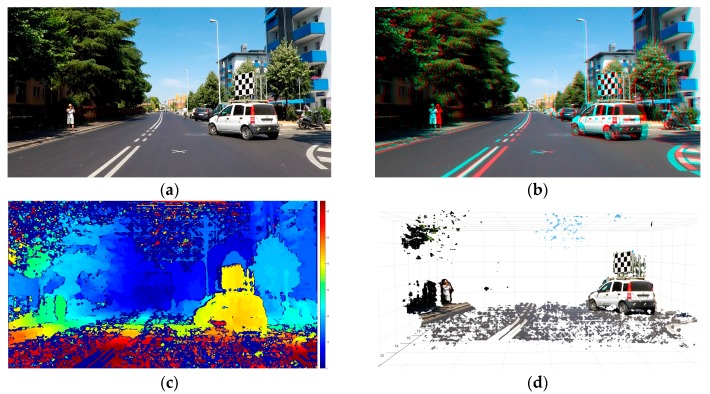
Analysis of the pre-crash scene (id90–InSAFE). (**a**) Rectified left picture of the long range stereo camera sensor; (**b**) The 3D anaglyph composed by the stereo frame; (**c**) Disparity Map (0 to 64d); (**d**) 3D reconstruction available for download (Appendix B).

For the present study we emulated one particular real-world motorcycle crash and tested the capabilities of our remote sensor based on stereo cameras to sense the conditions of the traffic scene in the neighborhood of the point where the collision became inevitable. In particular, the experiment consisted in remotely measuring the distance an orientation of the opponent vehicle (a passenger car) from the sensors in the scooter. For visual help, the reference point of deployment of M-AEB was marked on the road surface with a white “x” as visible in Figure 12a. The test was designed and executed so that when the scooter was located at this point the opponent car was located and oriented as it was in the real crash when the collision became inevitable.

In this experiment (pre-crash test) depicted in Figure 12, we analyzed whether our remote sensing approach was able to sense the condition of the traffic scene 1 second before the instance of unavoidable collision. If this is possible, an early prediction of the imminent collision may allow for a crash avoidance. One example of a possible preventive countermeasure is to send a stopping signal to the opponent car and trigger the Autonomous Emergency Braking (AEB) of the car, or simply honk to alert both car driver and motorcyclist of the dangerous situation.

In the 3D anaglyph of Figure 12b, the remote sensor is slightly tilted with respect to the road plane, as can be seen from the small vertical shifting of the features in the scene. For example, the vertical pixel misalignment in the stereo frame is noticeable in the “x” marking on the road, or in the corners of the asphalt marks located at the bottom right corner of the picture.

From the results shown (Figure 12c,d) the stereo camera sensor is able to accurately sense the condition of the traffic scene 1 s before of the instant of unavoidable collision. In addition to this, the sensing approach seems to be able to detect narrow obstacles including Vulnerable Road Users (VRUs), which is a challenging task for other automotive remote sensors (see Appendix D). In this case, a pedestrian was measured along the left sidewalk.

**Figure 13:**
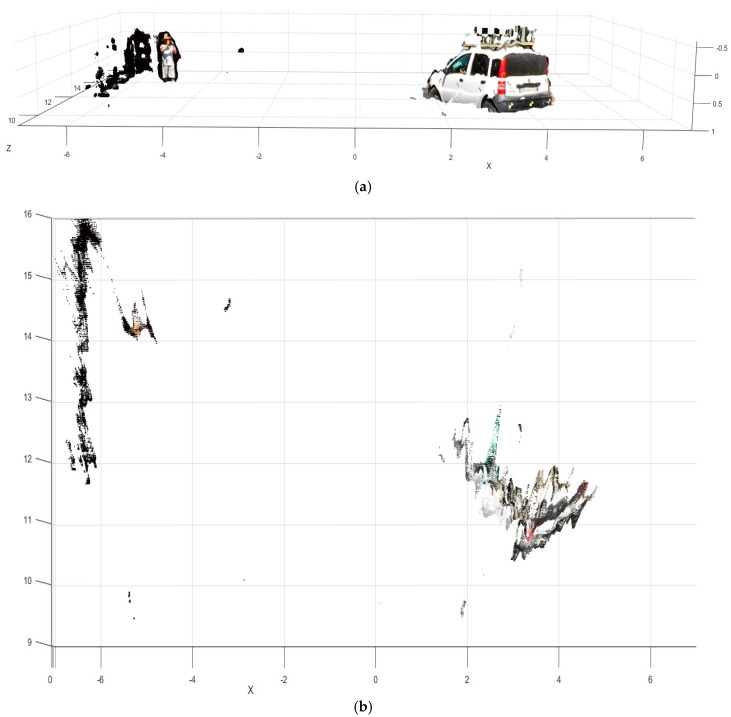
Detail of 3D reconstruction of the pre-crash scene (id90–InSAFE). (**a**) Cleaned 3D point cloud seen from the scooter point-of-view; (**b**) Cleaned top view representation of the pre-crash scene.

A clean 3D point cloud (extracting only the information from above the street up to several centimeters above the roof of the target cars) is presented in Figure 13.

**Figure 14:**
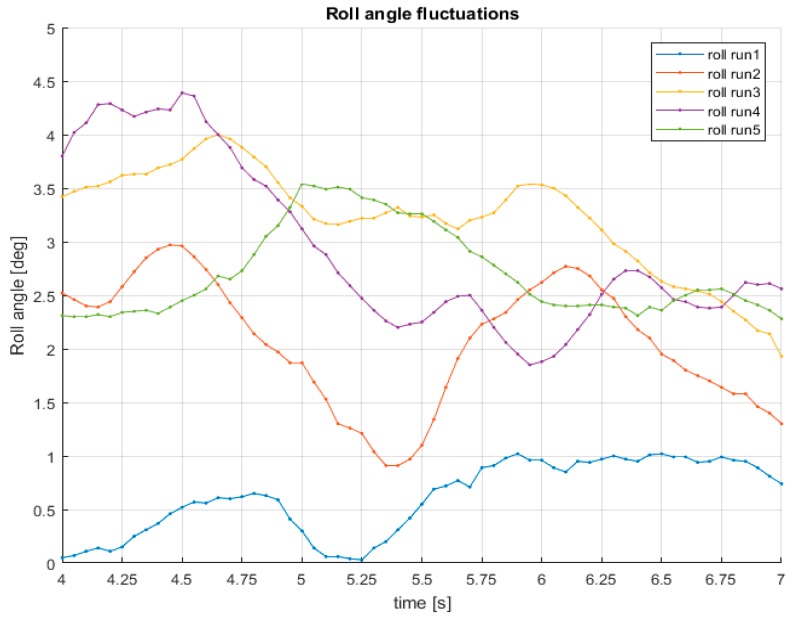
Roll angle fluctuations during 5 trials of the emulation of the motorcycle crash (id90–InSAFE).

It can be seen that part of the supporting frame of the satellite marker appears in the reconstruction. These results show that the remote sensing strategy is able to measure the car pose and narrow obstacles in real conditions even in the presence of roll angle fluctuations (Figure 14), that also can affect the operation of other automotive sensors as the LIDAR [24].

The temporal charts (Figure 14) presents the of roll angle fluctuations due to the dynamics of a tilting vehicle when traveling straight (note that the traveling surface is flat asphalt).

For the reproduction of our results and for algorithm benchmarking purposes, we provide an online image dataset with ground truth (Appendix C) belonging to one trial of the 1 s pre-crash test analyzed. The dataset contains 30 color stereo frames (raw data) and its 30 grayscale pre-rectified stereo frames. The pre-rectified frames were calculated according to the camera calibration values of Appendix A.

### 5.3. Quantification of the Integrity of the Disparity Map (during the Pre-Crash Test Sequence)

As explained in Section 3.5, a continuous recalibration of the system is a must for motorcycle safety application because the decalibration (calibration lost) of the stereo rig generates the corruption of the Disparity Map (DM). During the calculation of the DM, the SGM algorithm set to 0 pixels when it cannot find a correspondence patch in the epipolar line of the other image of the pair. These 0 values are holes in the DM and represent zones with not depth information. In our system, we empirically notice that it is not possible to calculate a proper 3D point cloud from the DM data when it contain less than 45% of matched pixels of each camera (belonging to the shortened frustum at the Horopter 10d).

**Figure 15:**
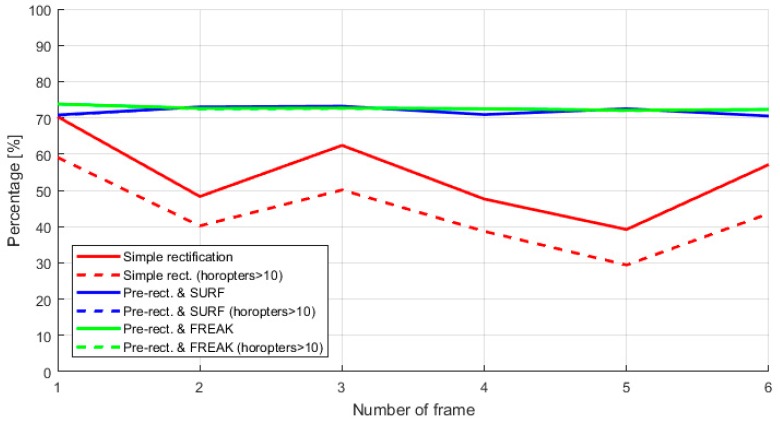
Chart showing the percentage of effective stereo frame used to calculate the Disparity Map (DM) during the first six neighboring frames (consecutive frames) of the 1 s pre-crash sequence. Below 45%, the number of reliable pixels is insufficient to compute the DM. The corresponding DMs for these six frames are presented in the Appendix C.

The Figure 15 presents a comparison of the percentage of pixels properly employed to compute the Disparity Map and obtain the depth of the scene. The comparison is done by normalizing the number of pixels on the Disparity Map computed and the number of pixels of an input imagen. The chart present in three different colors the results of the Simple rectification (static stereo calibration parameters), and the two strategies used for the re-calibration based in SURF and FREAK.

The continuous and dashed lines of the chart are used to distinguish between the totality of the pixels used to compute the DM and the most relevant set of disparities calculated, which are above of the Horopter 10d. For the two online re-calibration cases, the percentage of pixels used to calculate the DM appear to be stable around 70% to 74%, which is interesting and convenient. Also, in these cases the number of disparities below the Horopter 10d are negligible and consequently, the continuous and dashed lines in the chart are almost overlapping.

## 6. Discussion

The overall goal of our research activity was to contribute in making motorcycling safer by fostering the implementation of assistance technology. This technology can be transferred along to a variety of powered vehicles presenting a tilting motion, such as scooters, mopeds, mofas, and three- or four-wheeled tilting vehicles denominated Narrow Track Tilting Vehicles (NTTVs). 

This is important because the injury severity level in case of crashes is the biggest barrier for the societal adoption of tilting vehicles. In the case of motorcycle crashes, these types of road users are generally subjected to serious consequences for life. Motorcyclist are the 23% of the deaths on the world’s roads (World Health Organization, 2015), and they have 26-fold higher risk of death than those driving other types of vehicles (NHTSA USA, 2015).

Consequently, motorcycle safety systems to support people to avoid crashes must be enabled. Previous research identified a technological limitation on automotive remote sensors, for which these sensors cannot operate on a tilting vehicle [24]. To overcome this technological barrier we proposed an approach that utilizes camera-based sensors. We found these sensors suitable for the task thanks to their wide diagonal *FoV* (Field of View) and additional desirable features, such as resolution, lightweight, passive (no increase to electromagnetic pollution), low consumption and affordable cost.

In the present study, we targeted the development of a novel multi-focal stereo camera sensor to provide remote sensing able to operate under the constraints imposed by the motorcycle dynamics. The importance of the technological sensing solution we proposed relies on the potential to bridge the technological gap that causes the existing lack of rider assistance technologies for tilting vehicles. An example of application of sensing technologies for improved safety of motorcycles was provided with the emulation of a real motorcycle crash, as described here and in [110]. These tests conducted in real traffic conditions are part of the assessment of our remote sensor for the possible application in future ARAS (Advanced Rider Assistance Systems), for example a motorcycle application of Autonomous Emergency Braking (the so-called M-AEB).

In a camera-based perception system, the quality of the camera sensor is essential. The proliferation of mobile phones with camera sensors during the last decade, reinvents the way in which camera sensors work, as well as their performances. A proof of that is the ongoing standardization of the camera sensors for the automotive industry (Standard for Automotive System Image Quality-IEEE Project 2020), which started to work over the advanced draft of the current IEEE P1858 Standard for Camera Phone Image Quality. For related information, an overview of the image quality test for phone cameras is presented in [111].

Another important consideration is that the image sensor manufacturing technology is well below its limits. Image sensors use CMOS technologies that are at least 2 generations behind those of solid-state memories or digital integrated circuits. Thus, in the next few years several innovations in camera sensors are likely to take place. In particular, HDR imagers (High Dynamic Range camera sensors) are showing impressive capabilities using inexpensive technology [112]. Recent low-cost imagers feature a combination of RGB + IR (color and infrared) with controllable IR sensitivity [113]. This will make night vision cameras ubiquitous.

On the stereo algorithms side, the algorithms can calculate disparities in regions where there are no specularities or occlusions. In regions with low contrast or with high sensor noise, most implementations have difficulties. However, specific implementations allow 3D perception also in adverse weather like rain [85,114,115]. This result can be achieved by exploiting stereo confidence clues based in a probabilistic implementation (a Bayesian manner) of scene and temporal priors (prior knowledge of scene instants before) for the improvement of the stereo matching.

In addition, stereo cameras allow the discrimination of water on the road (possible slippery surfaces) by polarization light filters installed in each camera and machine-learning methods [116,117]. Another application for the detection of small road hazards was recently implemented by combining geometrical modeling and deep learning in autonomous cars context [97]. For further improvement, machine-learning researchers are also combining the visual information with the Disparity Map of stereo vison systems for an alternative three-dimensional understanding [73,118].

In our experiments, the performance of the sensor developed employing low-cost action cameras was satisfactory in a static setup. The sensor showed good potential for the application in advanced motorcycle safety systems as it was able to measure small targets sized 30 cm of height (traffic cones) from a distance up to 21 m and road curbs during the test in the traffic scenarios. This sensing capabilities are promising for motorcycle safety application, for which unexpected small obstacles in the travelling path or occasional slippery surfaces can cause serious consequences to the motorcyclist (destabilization, crashing and falling).

In this paper we mentioned several promising state-of-the-art solutions that are include machine-learning techniques. These technics require an intensive computational power that consumes significant amount of energy that will not be available in a tilting vehicles. Fortunately, recent embedded neural computers designed in a single chip (ASIC: Application-Specific Integrated Circuit), provide the capability to deploy certain Deep Neural Networks (DNNs) which a power consumption inferior to 1.2 watts. 

Other relevant aspect is that these ASICs have a Vision Processing Unit (VPU) to process camera data in real-time. This is important because all the algorithms used in this paper can be implemented in VPUs. These new chips and the upcoming improvements in camera sensors are promising technologies to make remote sensors camera-based, such as the one developed in this paper for motorcycle safety application.

## 7. Conclusions

The use of stereoscopic vision for motorcycle safety as the single remote sensor is possible, assuming clear visibility conditions (e.g., no fog, no rain, and no snow) in most urban crashes. The important aspect of this paper relies on the whole explanation of an affordable, lightweight, and low power consumption remote sensor able to 3D measure the environment from a tilting vehicle (the scooter demonstrator). Particular attention was also given to the operation of the sensor under fluctuations of the roll angle because, these fluctuations are present in the motorcycle dynamics, even when the vehicle is traveling straight and on flat asphalt (up to 4 degrees in the pre-crash scenario).

Additionally, the operation of the camera-based sensor was analyzed when the scooter cornering (up to 13 degrees in the turning maneuver). Rotation between reference coordinate frames, belonging to the host vehicle and the road obstacles, means that existing automotive sensors solutions do not work in the tilting vehicle domain [24].

This publication is the first integral approach to implement a remote sensor capable of enabling ADAS specifically for motorcycles with the potential to avoid crashes, the so-called ARAS: Advanced Rider Assistance Systems. This is an important step towards the concretization of preventive safety technologies in tilting vehicles. Vehicles that cannot offer important crash injury mitigation levels as passenger cars.

The feasibility study for the triggering of a motorcycle Autonomous Braking System (M-AEB) from our camera-based sensors started in [110] and continued in this research activity, showing additional encouraging results. The sensor was able to measure the distance to the opposite car up to 16 m of depth within the grid of 20 cm required by M-AEB during the pre-crash phase. This grid size was a target requirement of M-AEB to avoid false positives. In addition, as the expected intervention of M-AEB in urban scenarios is expected to be between 8 to 10 m of distance to the colliding car. Thus, the sensor developed fits comfortable the M-AEB requirements. In addition, the measurements performed up to 20 m shown a depth error inferior to 3%, which according to the European project INTERSAFE-2 is considered as a requirement for reliable driving assistance systems in passenger cars.

These results also seem prominent for the application of pose accuracy estimation methods that have the potential to accurate estimate the heading angle of the opponent vehicle. To this end, we provide material to the research community in the form of an image dataset for the 1 second pre-crash test (Appendix B), with the aim of supporting the development of safety systems for tilting vehicles. Our raw and pre-calibrated stereo frames, which requires online re-calibration, contain the effects of roll angle fluctuations typical of vehicles that present a tilting dynamics. Therefore, our data is unique in this sense.

During the experiments in the real traffic, we notice that the roughness of the road can negatively affect camera-based sensors performance. In particular, stereo camera sensors are more susceptible than monocular ones due to the extrinsic decalibration phenomena, which makes depth measurement unusable. As a consequence, we strongly suggest that future assessment of camera-based ARAS and ADAS, such as those of NCAP (New Car Assessment Program), should be carried out on different types of cobbled roads instead of just on the asphalt.

Finally, the satisfactory results achieved so far and presented in this paper warrant the validation of our remote sensor in a variety of different conditions to become a part of a safety system. For example: assessing the sensor and its algorithms under different levels of vibrations, the sensor response in tilting vehicles of different characteristics, the utilization of different kinds of imagers (i.e., High Dynamic Range, RGB+NIR, global shutter), and the assessment of the remote sensor in a wider range of motorcycle crash scenarios.

## Figures and Tables

**Table 1:** Specification of the stereo rigs and constitutive cameras.

Short-Range: Camera Pair III-IV			Long-Range: Camera Pair 2-1		
Baseline (cm)	15.0		Baseline (cm)	26.5	
Diag. *FoV* (°)	170		Diag. *FoV* (°)	90	
Depth Field (m)	1	8	Depth Field (m)	8	22
Resolution	1280	720	Resolution	1280	720
fps	30		fps	30	

**Table 2:** Range Field of the remote sensor calculated for the application on advanced safety systems.

	Camera Pair III-IV		Camera Pair 2-1	
	Far	Near	Far	Near
Disparity (pix)	17	118	24	57
Depth (m)	8.382	1.207	18.936	7.973
Depth discretization (m) & Sub-pixel = 1	0.465	0.010	0.757	0.137
Depth discretization (m) & Sub-pixel = 1/4	0.121	0.002	0.195	0.034

**Table 3:** Main values of the calibration of the stereo rigs for both measuring ranges.

Short-Range: Camera Pair III-IV				Long-Range: Camera Pair 2-1			
Baseline (cm)	14.9157			Baseline (cm)	26.4867		
Right Focal length (pix)	Left Focal length (pix)			Right Focal length (pix)	Left Focal length (pix)		
897.2688	897.6886	1027.5	1040.7	1715.3	1719.6	1712.6	1717.8

**Figure A1:**
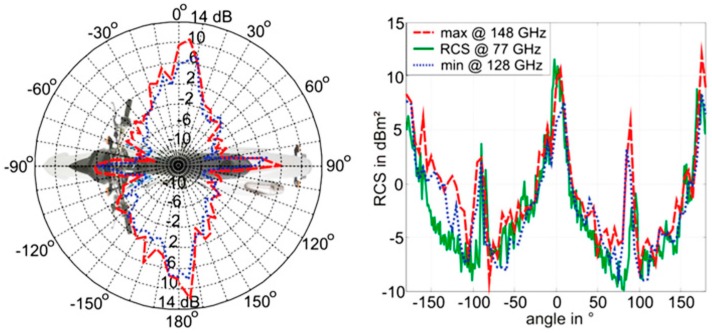
Radar Cross-Section of a motorcycle. Adapted from (Köhler et al. 2013) [119].

**Figure A2:**
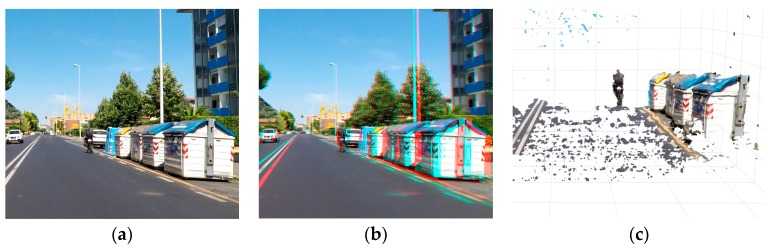
Example of the artificial depth interpretation of a real traffic scene thanks to the stereo camera sensor technology. (**a**) Left image acquired from a stereo camera pair onboard the instrumented scooter; (**b**) Anaglyph of the traffic scene, it can be see the scene in 3D by wearing color-coded glasses (red-blue ones); (**c**) 3D point cloud representation of the scene in which can be detected the location in the space of the cyclist and the garbage bins placed on the right side of the lane.

**Figure A3:**
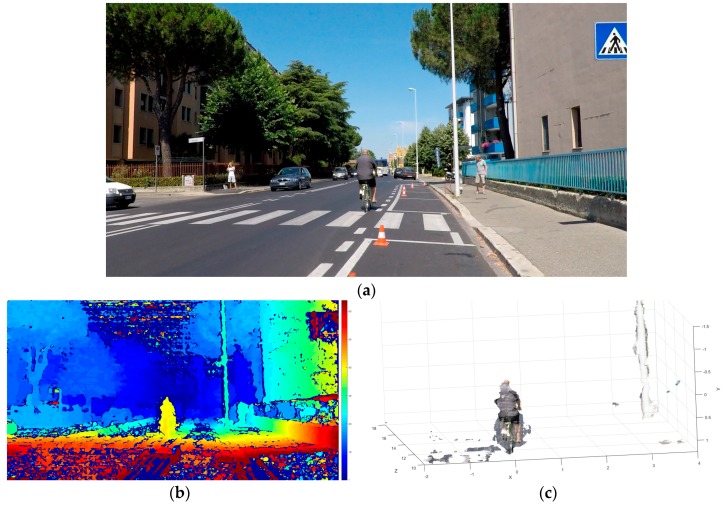
Sensing of a cyclist from a distance of 12 m. (**a**) Left image acquired from a stereo camera pair onboard the instrumented scooter; (**b**) Disparity map of the road scene (0 to 64d); (**c**) 3D point cloud representation of the scene in which can be detected the location in the space of the cyclist and the light pole about 5 m behind.

## Appendix A

Here we present the complete camera calibration values corresponding to both stereo camera rigs. The short-range stereo sensor is characterized for a wide *FoV* to sense the close surroundings of the tilting vehicle, while the long-range sensor is characterized for a narrow *FoV* to inspect the traffic lanes in front of it.

**Table A1:** Values of the calibration of the stereo rigs for both measuring ranges.

Short-Range: Camera Pair III-IV			Long-Range: Camera Pair 2−1		
Rotation Matrix of Camera IV w.r.t. III (rad)			Rotation Matrix of Camera 2 w.r.t. 1 (rad)		
1.0000	0.0090	−2.6874 × 10^−4^	1.0000	5.8039 × 10^−4^	0.0056
−0.0090	1.0000	0.0022	−5.2068 × 10^−4^	0.9999	−0.0106
2.8854 × 10^−4^	−0.0022	1.0000	−0.0056	0.0106	0.9999
Translation Vector of camera IV w.r.t. III (cm)			Translation Vector of camera 2 w.r.t. 1 (cm)		
−14.9157	−1.3716	−1.6951	−26.4867	0.1979	0.0216
Fundamental Matrix			Fundamental Matrix		
1.6930 × 10^−8^	1.8345 × 10^−6^	−0.0024	3.3535 × 10^−10^	−7.4253 × 10^−8^	1.5366 × 10^−4^
−1.8583 × 10^−6^	3.6987 × 10^−0.7^	0.1457	1.239 × 10^−7^	−9.5361 × 10^−8^	0.0154
0.0010	−0.1690	14.7106	−1.8817 × 10^−4^	−0.0153	0.0711
Essential Matrix			Essential Matrix		
0.0156	1.6920	−1.3753	9.8510 × 10^−4^	−0.2187	0.1955
−1.7353	0.3455	149.9148	0.3653	−0.2817	26.4836
0.0237	−1.49.9216	0.3303	−0.2132	−26.4851	−0.2804

**Table A2:** Values of the calibration of the right camera for both measuring ranges.

Short-Range: Camera Parameters III			Long-Range: Camera Parameters 2		
Radial distortion Vector			Radial distortion Vector		
−0.3545	0.2133		−0.2621	0.0772	
Tangential distortion Vector			Tangential distortion Vector		
3.3009 × 10^−4^	8.1015 × 10^−4^		0	0	
Focal length Vector (pix)			Focal length Vector (pix)		
897.2688	897.6886		1.7153 × 10^3^	1.7196 × 10^3^	
Principal point Vector (pix)			Principal point Vector (pix)		
1.0067 × 10^3^	557.7331		923.7204	535.7389	
Intrinsic Matrix (pix)			Intrinsic Matrix (pix)		
897.2688	0	0	1.7126 × 10^3^	0	0
0	897.6886	0	0	1.7178 × 10^3^	0
1.0067 × 10^3^	557.7331	1	950.4462	512.4862	1

**Table A3:** Values of the calibration of the left camera for both measuring ranges.

Short-Range: Camera Parameters IV			Long-Range: Camera Parameters 1		
Radial distortion Vector			Radial distortion Vector		
−0.3446	0.1757		−0.2652	0.0875	
Tangential distortion Vector			Tangential distortion Vector		
−7.0755 × 10^−4^	7.1722 × 10^−4^		0	0	
Focal length Vector (pix)			Focal length Vector (pix)		
1.0275 × 10^3^	1.0407 × 10^3^		1.7126 × 10^3^	1.7178 × 10^3^	
Principal point Vector (pix)			Principal point Vector (pix)		
989.0498	556.5230		950.4462	512.4862	
Intrinsic Matrix (pix)			Intrinsic Matrix (pix)		
1.0275 × 10^3^	0	0	1.7153 × 10^3^	0	0
0	1.0407 × 10^3^	0	0	1.7196 × 10^3^	0
989.0498	556.5230	1	923.7204	535.7389	1

## Appendix B

This appendix provide some data for visualization and experimentation purposes:

1. 3D reconstructed scenes: (*.pcd) files corresponding to the 3D point clouds acquired for the system which are shown in the publication. Files named “testFirenze*.pcd”.
2. Decalibration dataset: (*.png files):
  - A set of 30 color stereo pairs (raw data) corresponding to the pre-crash test sequence. Six files named “Original_crash_*.zip”.
  - A set of 30 rectified stereo pairs (pre-rectification according to the static camera calibration) corresponding to the pre-crash test sequence. Four files named “PreRect_crash_*.zip”.
  The data is available in the following repository:
  https://github.com/GusRep/StereoDecalibrationProblem_and_PointClouds
  The Wiki page associate to the repository is:
  https://github.com/GusRep/StereoDecalibrationProblem_and_PointClouds/wiki

The effects of decalibration problem and the qualitative results of the two strategies using to perform the online re-calibration is shown in Appendix C. In this case is presented the Disparity Map (DM) calculation over six consecutive frames and compared against the DM calculated for the re-calibrated stereo pairs. The satellite marker [93] can be used in the stereo frames to retrieve the ground truth heading angle of the vehicle and the instantaneous orientation of each camera.

## Appendix C

The following Disparity Maps (DMs) are organized by columns. The left column corresponds to the depth triangulation on the rectified images (Simple rectification) obtained from the static calibration (extrinsic + intrinsic parameters of the camera model), while the central and right columns corresponds to the re-calibration (second step of the online re-calibration).

In the 2nd frame corresponding to the static calibration it can be seen that the DM is corrupted due to the decalibration at this instance.

From the 4th and 5th frames of the static calibration cannot be measure the target due to the decalibration at these moments, while in the 6th frame the depth triangulation is possible again (borderline).

This short sequence of 6 consecutive frames make explicit the consequences of the dynamic stereo decalibration in the motorcycle application. As it can be seen in the two columns corresponding to the online re-calibration, both strategies present satisfactory results. Note that the color disparity scale between the online re-calibrated DMs is different to the scale of the fixed calibration to facilitate the visual comparison.

## Appendix D

Here we introduce the problem of lack of visibility of certain road users in order to present supplemental data recorded during the execution of the main experiments and our impressions about these cases.

### Accurate Narrow Obstacle Detections

Due to the encouraging observation of the proper identification of the light pole Figure 11b in and pedestrian in Figure 12d, we analyzed the possibility to identify narrow targets corresponding to Vulnerable Road Users (VRUs) in the recorded data of the previous experiments. VRUs, such as pedestrians, cyclist and motorcyclist, suffer the huge drawback in terms of road safety due to its difficult visibility in dynamic environments. In traffic safety this topic is knolled as “conspicuously problem” and affects to human car drivers but also to different automotive remote sensing technologies (e.g., LIDAR, and RADAR).

For the purposes of improved safety for VRUs, the European Union has carried out an extended measurement campaign to identify the Radar Cross-Section (RCS) of pedestrians [119] aiming to ensure that pedestrians can be seen by automotive radars. To present the magnitude of the problem, we interpret the results of a measurement of RCS corresponding to a motorcycle in Figure A1 [120].

The radar signal for the band of 79 GHz (77–81 GHz) is depicted in green, being the weakest of the three bands analyzed. These measurements show that the motorcycle returns a strong eco to the radar when it is placed with perfectly perpendicular alignment (0° and 180°). On the contrary, for different alignments the strength decrease considerably. For example, at 15° the signal is 10 times weaker with respect to the maximum signal received from the motorcycle, and it falls down to 40 times weaker for other orientations. Concerning the radar target visibility, it can be seen the poor visibility of the target for the measures realized to the front (between −50° and −130°) and rear side of the motorcycle (between 50° and 130°).

Back to our measurements, in Figure A2 we present a study case, in which a cyclist is traveling on the edge of the traveling lane of our instrumented scooter. The 3D anaglyph of the traffic scene (Figure A2b) allows to see the 3D content of the stereo frame while wearing anaglyph glasses, but without them it allows the verification of the proper image rectification process (linked to the camera calibration) and the measurement of the disparity range by a horizontal measurement (pixel counting) of different features of the scene.

The 3D reconstruction of the scene is presented in Figure A2c. As it can be seen the stereo vision system present enough horizontal and vertical special discrimination to identify the cross section of the bicycle and its cyclist. Additionally, the remote sensor on the scooter is also capable to measure the distance to the cyclist (narrow obstacle).

A different cyclist measured from the imaging system is presented in Figure A3. In this situation the instrumented scooter rider was halted in a parking spot and the cyclist passed just from the adjacent lane. In the disparity map (Figure A3b) it can be seen the clear presence of the cyclist in the Range Field of the sensor. To conclude, in Figure A3c is showed the 3D reconstruction of the cyclist and the traffic pole which is and object more narrow than the cyclist.
